# The World Checklist of Vascular Plants, a continuously updated resource for exploring global plant diversity

**DOI:** 10.1038/s41597-021-00997-6

**Published:** 2021-08-13

**Authors:** Rafaël Govaerts, Eimear Nic Lughadha, Nicholas Black, Robert Turner, Alan Paton

**Affiliations:** Royal Botanic Gardens, Kew, Richmond, UK

**Keywords:** Classification and taxonomy, Plant ecology, Biodiversity, Data publication and archiving, Data integration

## Abstract

The World Checklist of Vascular Plants (WCVP) is a comprehensive list of scientifically described plant species, compiled over four decades, from peer-reviewed literature, authoritative scientific databases, herbaria and observations, then reviewed by experts. It is a vital tool to facilitate plant diversity research, conservation and effective management, including sustainable use and equitable sharing of benefits. To maximise utility, such lists should be accessible, explicitly evidence-based, transparent, expert-reviewed, and regularly updated, incorporating new evidence and emerging scientific consensus. WCVP largely meets these criteria, being continuously updated and freely available online. Users can browse, search, or download a user-defined subset of accepted species with corresponding synonyms and bibliographic details, or a date-stamped full dataset. To facilitate appropriate data reuse by individual researchers and global initiatives including Global Biodiversity Information Facility, Catalogue of Life and World Flora Online, we document data collation and review processes, the underlying data structure, and the international data standards and technical validation that ensure data quality and integrity. We also address the questions most frequently received from users.

## Abstract (summary)

**Table:** 

Measurement(s)	Vascular Plant • Species
Technology Type(s)	digital curation
Sample Characteristic - Organism	Tracheophyta
Sample Characteristic - Location	global

Machine-accessible metadata file describing the reported data: 10.6084/m9.figshare.15035046

## Background & Summary

The World Checklist of Vascular Plants (WCVP, http://wcvp.science.kew.org/) is a sustainable, curated, global consensus view of all known vascular plant species (flowering plants, conifers, cycads, ferns, clubmosses and firmosses). It is derived from names and taxonomic concept resources managed at the Royal Botanic Gardens, Kew (hereafter Kew) by reconciling names to taxon concepts, to produce a comprehensive taxonomic compilation for vascular plants. By 16th April 2021, the WCVP database included 1,383,297 plant names, 996,093 at species level, representing 342,953 accepted vascular plant species. For each accepted species, WCVP presents bibliographic information (author, place and date of publication) and taxon concept information comprising comprehensive synonymy and family placement following standard references for angiosperms^1^ and for other groups^2^.

The foundation for WCVP is the International Plant Names Index (IPNI, http://www.ipni.org), an authoritative source of objective nomenclatural data which collates and indexes nomenclatural acts (including spelling, author(s), type(s), place and date of publication). Building on these nomenclatural facts, WCVP adds taxonomic data, recording each name as accepted, a synonym or unplaced. The taxonomy is sourced primarily from botanical literature but also from herbarium specimens, observations and experts. Names absent from IPNI are added. Completed family treatments are reviewed by experts. Most families with completed expert review are already published in World Checklist of Selected Plant Families (WCSP, http://wcsp.science.kew.org/), providing taxon concept data for 200 vascular plant families. Taxonomic compilation of WCVP is now complete and taxon concept data in families not published via WCSP (totalling 203,571 species) are currently being reviewed by experts. WCVP is edited daily and updated weekly. In 2019, over 2,000 feedback e-mails were received, and c. 500,000 individual edits made to WCVP data.

References supporting taxonomic status decisions in WCVP, publications disagreeing with such decisions, and those presenting alternative taxonomies, are recorded. WCVP also records geographic distribution by botanical country^3^ for all taxa. Geographic data are near-complete but omitted from the published dataset. We plan to complete geographic data for WCVP for publication in 2021. Thereafter WCSP will be discontinued. WCVP will provide access to geographic data and taxonomic references.

Some original data for WCVP were provided by generous collaborators (see Methods). WCVP aims to present a global consensus view of current plant taxonomy at species level, reflecting recent publications while incorporating opinions of plant taxonomists around the world.

The WCVP differs from other global plant lists (e.g. *The Leipzig Catalogue of Vascular Plants*^4^, *The Plant List 1.1* (TPL1.1, www.theplantlist.org), and WorldPlants (www.worldplants.de) in many respects (Table 1); here we list the most important. The status of each name is considered by an expert compiler, avoiding errors introduced by machine-generated taxonomic links. Binomials that do not refer to a species concept, do not conform to the *International Code of Nomenclature*^5^ (ICN), or belong to a non-accepted genus are listed as unplaced. Other global plant lists often accept such names, even those that do not conform to ICN and should not be used. Within each family, taxon concepts are reviewed by taxonomic experts. References to literature or communications supporting taxonomic changes are linked to individual records. WCVP is edited daily and updated weekly, reflecting user feedback and new publications to build a global consensus.

**Table 1:** Comparison of three current comprehensive online lists of vascular plants: Leipzig Catalogue of Vascular Plants (LCVP), World Checklist of Vascular Plants (WCVP) and WorldPlants.

	LCVP	WCVP	WorldPlants
**Access**			
Website/portal	No (planned)	https://wcvp.science.kew.org/	www.worldplants.de
Latest download*	5 July 2020	16 March 2021	No downloads
Full data download	https://idata.idiv.de/ddm/Data/ShowData/1806	http://sftp.kew.org/pub/data-repositories/WCVP/	—
Also available via:	—	GBIF, Catalogue of Life p.p 35%, WCSP, POWO, WFO	GBIF, Catalogue of Life p.p. 60%
Data management format	lcvplants package database	ACCESS and SYBASE database	Text file
**Update & review**			
Frequency	Constant updates planned, and a new version to be released every 2–3 years	Weekly via website	Website 2(−4) per month
Expert reviewed?	no	yes	no
Taxonomic references	Based on existing databases and an additional 4,500 publications	References to all taxonomic decisions and geographic distributions are captured and attached to individual records. A total of 9,464 references are used.	No direct links to taxonomic works except for protologues. Cross-checks against generic revisions and single taxonomic papers planned. No full list of the references used is published.
Vascular Plant totals**			
Total names	1,315,562	1,383,297	Not available
Accepted names (species)	351,180	342,953	352,048
Synonyms	846,279	925,561	Not available
Doubtful/unplaced/unresolved names	63,072	50,986	Not available
Families accepted	564	452	525
Genera accepted	13,460	13,778	14,361

The Plant List (TPL 1.1) is not included in this comparison because it is no longer maintained (last updated in 2013, based on 2012 data).

*Dates checked on 16 April 2021.

**Record numbers in this section derive from the most up-to-date versions available at the time of Data Descriptor preparation: LCVP 5 July 2020; WCVP 16 April 2021; WorldPlants 14 March 2021.

The WCVP serves as the taxonomic index to *Plants of the World Online* (POWO, www.plantsoftheworldonline.org). Subsets of WCVP are published in *Catalogue of Life* and the dataset has been submitted to *GBIF/Catalogue of Life Plus*^6^. WCVP is replacing TPL1.1. as the taxonomic default for *World Flora Online* (WFO, www.worldfloraonline.org), providing taxon concept data for taxa not provided by WFO’s Taxonomic Expert Networks (TENs)^7^.

Numerous academic publications have used WCVP data for research at geographic scales from global studies to those focusing on single megadiverse countries or individual islands. Research topics addressed range from biogeography and conservation to phylogenomics and phytochemistry (Online-only Table 1). Potential reuse of the data extends far beyond academic publications: WCVP data expedite preparation of national checklists^8^, stimulate extinction risk assessments^9,10^ and support implementation of international conventions and policies^11,12^.

The WCVP dataset is available from a simple, intuitive web interface that facilitates plant name searching, browsing the taxonomic hierarchy (up to family) or user-defined downloads. Data are updated weekly. Snapshots of the whole dataset are archived at GBIF.org and the Kew FTP server (See Data Records) and free to download.

## Methods

The compilation, editing and review of WCVP spanned the digital revolution. Therefore, the format in which the data were stored and distributed, the format in which data were obtained and accessed changed radically over time. However, the key elements and core workflows stayed largely the same. Here we present an overview of these workflows and then provide more detail on each workflow in turn, before describing the approaches to standardization, taxon acceptance, alternative taxonomies and international collaboration adopted during the preparation of what became the WCVP dataset.

### Overview of workflows

Four main workflows operated in parallel:

(i) The A-Z workflow in which each name was mapped to a taxon concept, if possible, and the correct name for each accepted taxon concept identified, the others being recorded as synonyms of an accepted name or unplaced (when not mapped).
(ii) The family review workflow whereby, once a family checklist was complete in draft, the checklist or portions thereof were sent for expert review by taxonomists with relevant expertise, whether at Kew or around the world. Once feedback from expert review had been considered, and incorporated where appropriate, family treatments were published on the WCSP website.
(iii) The geographic workflow focuses primarily on recording the global distribution of each accepted taxon in terms of its presence in the botanical countries of the world^3^^.^
(iv) The update workflow is a continuous process of updating the dataset and incorporating new information gleaned from new publications, directly or via IPNI, as well as from user feedback and expert review focused on particular subsets of the data (*e.g*. genera).

The parallel operation of these four workflows over decades resulted in data being checked and rechecked multiple times. For example, the widespread grass *Poa annua* has 264 country codes added and 67 references listed, indicating that the record was checked at least 67 times. All workflows use as a starting point standardised nomenclatural data from IPNI or by screening the literature during the workflows and adding standardised names missing from IPNI as they are encountered. This process is described under the A-Z workflow and in the Standards Used section. All workflows involve taxonomic decision-making processes described in the Taxon Acceptance section.

### The A-Z workflow in detail

The A-Z workflow started in 1988 and was completed on 4 December 2019. Name data from *Index Kewensis* (IK), which in 2000 was incorporated into IPNI, was initially retyped into a Firefox database and digitally copied from 1995. These raw data contained different formats reflecting non-standard formatting throughout IK’s history and lacked many dates of publication. The data were therefore first standardised using the standards described below before they were imported. In the early years, the coverage of the name data was still incomplete as names were added from IK in five batches between 1995 and 2008, each batch being standardised before being added to WCVP. Compilation began with the genus Aa Rchb.f. and continued alphabetically through all the genera. The relevant literature on the genus was then consulted at Botanic Garden Meise and Kew to ascertain the taxonomic status of each name (see below) and to add any distribution data encountered, as well as some 190,000 names missing from IK/IPNI. The latter step was particularly important for infraspecific names, as these were not systematically recorded in IK before 1971. During the compilation process, names missing from WCVP are added when encountered and therefore the infraspecific names should be largely complete for those in current use. In parallel, infraspecific names from other databases have been imported and some historic literature important to particular families has been screened for all names. During this process duplicates were removed and names were also checked to make sure they complied with the ICN^5^. Despite the above, many validly published infraspecific names are still missing from WCVP, especially historic names.

Each name was assigned one of three basic taxonomic statuses: Accepted, Synonym or Unplaced.

If a name was accepted in a publication as a distinct species with a published species concept, then the name was given the status ‘Accepted’ and geographic distribution data were added from that source. The database differentiates two different kinds of accepted name, the most frequently assigned accepted name status is given to native plants that occur in the wild while the “Artificial Hybrid” status is assigned to names that are correct and can be used for cultivated or naturalised taxa that are either man-made and do not occur in the wild (not wild plants) or those that may have a combination of natural and human-influenced components such spontaneous hybrids occurring in gardens or between native and introduced taxa.

If a name was listed as a synonym in a publication or in the original volume of IK, the status given would be “Synonym” and the name would be linked to the published accepted name. Several different types of synonyms are recorded, depending on their nomenclatural status as defined by the ICN: legitimate synonyms, illegitimate synonyms, not validly published synonyms, orthographic variants and misapplied.

If a name was not encountered in any of the literature consulted it was assigned “Unplaced” status. This status is also used for names that would be accepted but for the fact that they are illegitimate or not validly published under the ICN and therefore cannot be used for taxa that should be accepted but do not have a correct name in an accepted genus. The most common occurrence of this last case are names published in genera that are not accepted in WCVP, but for which a validly published combination in an accepted genus does not exist. Distribution is also added for unplaced names as they may relate to distinct species concepts and may become accepted under a legitimate, validly published name in future or can be used as an aid to resolve them at regional level.

### The Family Review workflow in detail

The Family review workflow started in 1994 when RG was first employed by RBG, Kew. The idea is simple, a basic checklist is completed for a particular family. Relevant parts are then sent for review by taxonomic experts based in many different institutes worldwide. Recommended changes are then incorporated, and the checklist is published as a book and/or online on WCSP.

The families selected as World Checklist foci in the first instance were chosen because Kew had a particular research interest in that family, and expertise acquired over decades of research could be captured before key senior scientists retired (e.g. *World Checklist of Euphorbiaceae*^13^). Publication of a global treatment of a family at genus level also prompted and facilitated some family checklists. For example, the availability of a genus level classification of palms^14^ facilitated compilation of the palm checklist originally published as part of WCSP and as a book^15^, which in turn formed the basis for the online resource, *Palmweb* (www.palmweb.org). Similarly, a genus level treatment of Sapotaceae^16^ facilitated production of *the World Checklist of Sapotaceae*^17^ which is incorporated into the online *Sapotaceae Resource Centre* (https://padme.rbge.org.uk/Sapotaceae/data)).

As part of the review workflow, the full synonymy of each taxon concept is carefully checked to make sure the oldest available correct name is accepted for the concept. Sometimes a widely used name was accepted, even though an apparent earlier synonym was found. There are currently some 300 such synonyms indicated as possible earlier names pending further research. If these are confirmed as earlier names following further research it may be appropriate to consider formal rejection of these 300 names, in the interests of nomenclatural stability.

Approaches to family review varied because each plant family tends to have a particular expert community (or sometimes more than one) who collaborate best in different ways. For some families, experts were sent checklists of genera they requested to review, while for other families, such as Myrtaceae^18^, a workshop was held where all available experts were invited to put together a review strategy. For large families, such as Rubiaceae, experts agreeing to review the whole checklist worked through stacks of printout more than 60 cm high. All these diverse review approaches worked well and much improved the basic checklist. Once the review was completed, the family was added to the WCSP website and thereafter updated via the update workflow below.

### The Geographic workflow in detail

The geographic workflow started in 1995, when data were first imported electronically into the WCVP database from the IK database at RBG, Kew. Data entry via this workflow is continuing and is expected to be completed by mid 2021.

This workflow primarily focuses on adding the geographic data from published Floras and regional checklists. Such publications differ in geographic scope from individual protected areas to continental works published over decades. Over the years, the geographic workflow checked first Europe, then Africa, Southern America, Northern America, Asia, Subantarctic, Pacific and is currently finishing the floras of India and Australasia for the families in review. Geographic distribution information was captured using the standard codes at the level of Botanical Country (level 3) of the *World Geographical Scheme for Recording Plant Distributions*^6^ (hereafter WGSRPD).

In addition to the geographic distribution information that was added for accepted taxa, synonymy and missing infraspecific names were also added from those publications in order to speed up the A-Z workflow. Lifeform^19^, and climate zones data (see Standards Used below) for accepted species are also added at this stage, although this data is currently published only for families included in WCSP due to the constraints of current data platforms. When the geographical codes added to a record were deemed to be complete or nearly so, the geography was also added in words, which could be very specific for local endemics or very general for widespread species. The wording of the text would, as far as possible, use the same wording as used in the WGSRPD or a combination thereof. So, a species occurring in BZE (Northeast Brazil) and BZL (Southeast Brazil) would be reported to occur in E. Brazil (Eastern Brazil).

### The Update workflow in detail

The update workflow started in 1988, at the same time as the A-Z workflow and will continue as long as WCVP is maintained. The update workflow comprises three parts, weekly updates to the WCVP data available online, incorporation of user feedback and annual import of names added to IPNI in the previous year.

Every day new scientific insights are published and once a week all new journals and books that arrive in RG’s institute are screened and new data incorporated into WCVP. This was first done in the Belgian Botanic Garden library and from 1994 in the library of the Royal Botanic Gardens, Kew. There is also a proliferation of new online journals and eBooks, many of which come to our attention only if authors notify us of their publications. Automation of this literature review process has not been attempted to date due to: (i) the challenges inherent in detecting new synonymy or genuine nomenclatural corrections, as opposed to newly published names which are clearly indicated in compliance with the ICN; (ii) the need for a single process to ensure systematic coverage of the scientific literature; (iii) resource limitations.

The second source of updates comes from the daily stream of emails from users. Some 2,000 emails are received annually, and much improve the data. We aim to address all feedback within two weeks, although some queries requiring further discussion and library consultation may take longer and often involve discussions with the person sending the feedback. We also get requests to review particular genera from experts to whom we send data for review and then amend the database accordingly.

The third source of updates is names data downloaded from IPNI. Early in each calendar year, the scientific names added to IPNI in the previous year are imported manually to WCVP. They are then edited by adding taxonomic status and geography to each record in line with other workflows. In parallel, work is currently ongoing to reconcile all the names stored in the IPNI database with those stored in WCVP so eventually both datasets can share the same permanent IPNI identifiers.

Updates from the above sources become available to WCVP and POWO users on a weekly basis when the names data accessible from the WCVP web portal are updated. The full data download files are refreshed less frequently (currently every few months) because this requires a manual process, pending development of new infrastructure, including an Application Programming Interface.

### Standards used

From the outset of compilation work internationally agreed standards have been used to standardise the data. Originally, the database followed the fields proposed by the International Transfer Format for Botanic Garden Records^20^. This has proven to be important when migrating data to new IT systems and exchanging data with partners. Some of the fields have, over time, become more atomised but the information distributed across them is largely unchanged.

For nomenclatural terms and abbreviations and of course for nomenclatural practice in general, we follow the ICN^5^

Most of the other standards used to standardize data in the published WCVP dataset are recognised by Biodiversity Information Standards (www.tdwg.org):

- For the authors of plant names, we use *Authors of Plant Names*^21^ now maintained by IPNI. This standard is widely used and obligatory in many scientific journals.
- For journals, the second edition of *Botanico-Periodicum-Huntianum (BPH-2)* is used^22^.
- For books published until 1945, the second edition of *Taxonomic Literature* (*TL-2*)^23^ is used.
- For publications not in TL-2 and for books published after 1945, we follow the standard forms from the IPNI Publication Database which is continuously maintained.

For the additional data in WCVP, not included in the published dataset, the following standards are applied:

- For the geographical data we use *World Geographical Scheme for Recording Plant Distributions*^3^ with some minor changes for countries that have recently changed name, e.g. Swaziland for which we now use Eswatini.
- For the life form data, we follow the system originally proposed by Raunkiær^19^
- Climate zones: Alpine & Arctic, Temperate, Subtropical, Desert, Seasonally Dry Tropical and Wet Tropical used as consistent terminology to summarize the published habitat information from the resources used to construct each species concept.

### Taxon acceptance and species concepts

The basic rule of species acceptance in WCVP is very simple; we follow the latest published species concept unless experts advise us otherwise. Of course, anyone familiar with plant taxonomy will immediately realise that taxon acceptance is rarely that straightforward. It is however very important to make a distinction between acceptance in the different taxonomic ranks represented in WCVP (Family, Genus, Species, Infraspecifics). WCVP is primarily a list of species concepts. Taxa at other ranks are not the primary focus, not least because there will always be alternative classifications for stable species concepts. However, since full synonymy is provided, users can easily find the correct name if they prefer to use different generic or infraspecific concepts.

Although there is a pervasive impression that taxonomy is ever-changing and that alternative taxonomies are commonplace, this not our overall experience^24^. This perception may have some truth at generic level but from our experience there are very few current alternative species concepts supported by multiple scientists. Even at generic level alternative taxonomies are perhaps less problematic than is generally perceived, as shown for example by Vorontsova & Simon who suggest that up to 90% of names will remain unchanged when implementing a monophyletic classification for grasses^25^. Overall, there is striking consensus at species level, especially as for some groups there are very few if any active taxonomists. Internet searches may sometimes give the impression that multiple species concepts are accepted at the same time, but of course this is merely because older data are neither removed nor updated. It is therefore very important when using online resources to check the date on which a species concept was last updated or which published taxonomy is followed, because even a suppressed name such as *Solanum ferox* L. can still be found as seemingly accepted online.

Species acceptance in WCVP should be seen as a process rather than a one-off decision to which we adhere no matter what. As explained above under workflows, different publications are used to add the geography and create the species concept and they may not be screened in chronological order. In principle, during compilation we follow the latest published taxonomy and prioritise global accounts over local ones. These two principles are generally sufficient to provide species concepts for the vast majority of names. For the minority of cases, for which no recent taxonomic treatment exists and different current Floras adopt apparently different species concepts, then the situation is examined more closely: we try to find published peer-reviewed papers that include a phylogenetic treatment of the taxon, even if the paper lacks a formal taxonomic component, or we contact experts in the group to request resolution. Where uncertainty remains, then we generally default to retaining the existing taxon concepts rather than merging them without sufficient scientific evidence. All the initial species concepts adopted during collation then undergo the expert review process which will confirm or refine them.

For flowering plant families we follow APG IV^1^ and for conifers and ferns we follow *Plants of the World*^2^ including some recently published minor changes and additions^26^, for example. For genera we primarily follow global classifications where published (e.g. *Legumes of the World*^27^ and updates for the genera of Fabaceae, then partial generic classifications if such exist and *Plants of the World*^2^ for genera of which no recent published classification exists.) The generic classifications are also fine-tuned during the review process which is led by specialists in the relevant groups who may have more current, sometimes unpublished data to hand. Infraspecific taxa are accepted in a similar way as species concepts, they do however have the additional complication that for a large part of botanical history, most cultivars were given scientific names. As WCVP only records naturally evolved taxa, names applying to these mutations or human selections are synonymised under the species to which these mutations or cultivars belong. The epithets may be available under the *International Code of Nomenclature for Cultivated Plants*^28^, and appropriate cultivar names should be used as set out under that code.

### Alternative taxonomies

Botanists, in particular, ask the question if WCVP shows alternative taxonomies. Although this is perceived as being a major issue, we have never found this an issue in the review process or in general use. First, we should emphasize that WCVP is primarily a list of published species concepts and that currently most disagreements are about genera (See also Taxon Acceptance and Species Concepts above). WCVP lists all synonyms and therefore users are, of course, free to use a name in a different genus for the WCVP species concept. For genera we normally follow a published account that involved most of the experts of that group. For example, WCVP follows *Genera Orchidacearum*^29^ and subsequent volumes for the generic concepts in the family Orchidaceae with minor changes being made subsequently through discussions and feedback from the authors. The main advantage of following a particular account is that the generic circumscriptions are consistent and based on shared scientific evidence.

WCVP reflects alternative taxonomies in the references cited for each record, which are available through the links on the WCVP website to POWO. It became possible from 2003 onwards to add references for each name and each geographical record. Currently a total of 9,145 publications have been used and cited. When taxonomic changes are made to WCVP, a reference is added so users can see the publications or communications on which this change was based. It is important to make clear that (i) such references are only added to names or synonyms explicitly cited in the publication added and (ii) that the protologue (the work in which the name was originally published) is also a reference and this is included for each name. As a result, for some taxonomic decisions, the reference to the taxonomic work which provides the evidence for the decision may not appear in the record of each name affected by that decision, but only in a linked name record.

Although, over time, many species concepts have changed, in the here and now there are few competing species concepts where there is genuine disagreement with scientific evidence. While it may still be desirable to show current alternative taxonomies, we consider citing references to the competing view as the most objective and practical way to do this.

### International collaboration

As noted above, WCVP relies on collaborators around the world. 155 reviewers from 22 countries have been directly involved in expert review of the data for completed families and many others are currently reviewing data. WCVP also has a close relationship with several monographic resources in addition to the family level checklists mentioned above, including *Grassbase* (www.kew.org/data/grasses-db/index.htm), The Zingiberaceae Resource Centre (https://padme.rbge.org.uk/ZRC/), Cate Araceae (http://cate-araceae.myspecies.info/) and Palmweb (www.palmweb.org), and the Leguminosae^30^. WCVP also collaborates with floristic initiatives such as the *Catálogo de plantas e fungos do Brasil*^8^, *Euro* + *Med Plantbase* (http://ww2.bgbm.org/EuroPlusMed/), and World Flora Online^13^. Collaboration with horticultural data providers is strong too, including the *International Daffodil Register* (https://apps.rhs.org.uk/horticulturaldatabase/daffodilregister/daffsearch.asp) and the *Classified List and International Orchid Register* (https://apps.rhs.org.uk/horticulturaldatabase/orchidregister/orchidregister.asp).

WCVP has contributed data to the Catalogue of Life (CoL) and now provides 35% of vascular plant CoL content^31^. With increasing collaboration between CoL and GBIF in the CoL+ project^6^ and support of the World Flora on-line community^7^, CoL+ is likely to become the central hub for access to community-supported consensus taxonomic species lists covering all life. WCVP will provide its data through these initiatives, and will both work with TENs and provide taxon concept data for taxa not covered by any TEN. WCVP is already a baseline resource for TENs for certain plant groups (e.g. palms, legumes) and a source of update information for other TENs. In the case of the palm family, the WFO TEN has been closely involved since the compilation phase of WCVP and WCVP contributes the palm taxonomic data to WFO. The legume community is actively editing and commenting on current WCVP content. For other families e.g. Zingiberaceae, the TEN and WCVP run in parallel and data is frequently exchanged between the TEN and the WCVP editor. Thus the nature of the relationships vary, and in many cases they are still evolving, but clearly have the potential to be mutually beneficial and synergistic, with feedback from TENs helping to update WCVP records. WCVP downloads and website can assist any TEN in the task of routine curation and monitoring the addition of new names. WCVP welcomes collaboration with any TEN. It is envisaged that, eventually, TENs will cover all vascular plant groups and consensus content will flow from TENs through WFO to GBIF and CoL+. However, at the moment only 25% of vascular plant species are covered by the 29 TENs. Hence, the WCVP is a vital resource for updating and supporting the developing TENs network to achieve their vision.

### Principles for creating a single authoritative list of the world’s species

A recent paper presented ten principles that can underpin a governance framework for species lists^32^. Although the origins of WCVP predate this publication by decades, these principles have also underpinned the creation and governance of WCVP. We present a summary in Table 2.

**Table 2:** Ten principles which could underpin a governance framework for global species lists (Garnett *et al*.)^32^ and the ways in which WCVP already embodies them.

Garnett *et al*. principle	WCVP implementation
(i) the species list must be based on science and free from nontaxonomic considerations and interference.	A draft checklist based on scientific publications (peer-reviewed papers and books), is enhanced by expert review.
(ii) governance of the species list must aim for community support and use.	Work was often instigated by the scientific community working on a particular family. WCVP lists continue to be used by the scientific community in their outputs (see Table 1) and they continue to provide feedback to WCVP (see Background & Summary).
(iii) all decisions about list composition must be transparent.	All decisions on taxonomic status and geographic distribution made since 2003 are referenced. These are visible in *World Checklist of Selected Plant Families* and *Plants of the World Online* and will be available in WCVP once geographic data has been completed, maximising transparency.
(iv) the governance of validated lists of species is separate from the governance of the names of taxa	Names are sourced from the nomenclator the *International Plant Names Index*, thereby also providing stable LSID’s. A nomenclatural expert is part of the team. Nomenclatural considerations are separate from the taxonomic decisions taken on the same dataset.
(v) governance of lists of accepted species must not constrain academic freedom.	WCVP attempts a consensus view, but emphasizes that is just a snapshot in time. It changes continuously to incorporate new findings. It provides references to alternative taxonomies as well as all the names required to implement such an alternative taxonomy. Regular updates are made available to users who are free to use the content in forms that diverge from the taxonomic framework provided.
(vi) the set of criteria considered sufficient to recognise species boundaries may appropriately vary among different taxonomic groups but should be consistent when possible.	Consistency of application of species concepts within families is achieved by having a family-level focus for review, which involves expert input. Maintaining a central editing team collating the dataset as a whole maximises overall consistency.
(vii) a global list must balance conflicting needs for currency and stability by having archived versions.	Dated, archived versions of the data can be downloaded from the website.
(viii) contributors need appropriate recognition.	WCVP recognises contributors at multiple levels: individual corrections by experts are referenced as personal communications; a dedicated part of the website gives a full list of contributors; all contributors to a completed family checklists are referenced in the record of each name in that family
(ix) list content should be traceable.	In WCVP, both the protologue and the references used to make taxonomic decisions are cited via the link to *Plants of the World online*.
(x) a global listing process needs both to encompass global diversity and to accommodate local knowledge of that diversity.	The review process in WCVP is family-based in order to provide a globally consistent overview. However, many of the experts contributing to a family checklist choose to review subsets of the data most relevant to their expertise e.g. individual genera or taxa confined to particular regions. This approach maximises the opportunity to incorporate local knowledge. We also contribute data to regional databases who give feedback on their particular regions. As each species record includes detailed distribution information, it is easy to use regional filters to extract data for review and use by local experts.

## Data Records

The download of the data set is available via the GBIF.org repository^33^ under a CC-BY 4.0 Licence in a Darwin Core Archive File (10.15468/6h8ucr see Table 3). This data set, prepared on 16^th^ Mar. 2021, comprises 1,190,715 records, including 400,341 accepted names, 744,145 synonyms, 3610 artificial hybrids and 42,619 unplaced names.

**Table 3:** Content of data downloads available from World Checklist of Vascular Plants from GBIF.org and the Kew FTP server.

GBIF (DwC) Field Name	Kew FTP Field name	Note
taxonID	kew_id	International Plant Name Index (IPNI) identifier.
family	family	The name of the family to which the taxon belongs. (The highest rank at which names are presented in WCVP)
genus	genus	The name of the genus to which the record refers.
specificEpithet	species	The species epithet which is combined with the genus name to make a binomial name for a species
infraSpecificEpithet	infraspecies	The infraspecific epithet which is combined with a binomial to make a trinomial name at infraspecific rank, most commonly a subspecies or variety
scientificName	taxon_name	Concatenation of genus with species and, where applicable, infraspecific epithets to make a binomial or trinomial name
scientificNameAuthorship	authors	The author or authors responsible for publication of the scientific name
taxonRank	rank	The level in the taxonomic hierarchy where the taxon name fits
taxonomicStatus	taxonomic_status	Indication of taxonomic opinion re the name: accepted, synonym or unplaced
acceptedNameUsageID	accepted_kew_id	IPNI identifier of accepted name
	accepted_name	Accepted name (same as taxon name if taxonomic status is accepted).
	accepted_authors	Authors of accepted name
parentNameUsageID	parent_kew_id	ID of the record at the next level up in the taxonomic hierarchy
	parent_name	Name of the taxon in the next level up in the taxonomic hierarchy
	parent_authors	Authors of the parent name
	reviewed	Flag to indicate if the name has been peer reviewed.

Field names (which appear as field headers in the download) and explanatory notes on their content.

**Fig. 1:**
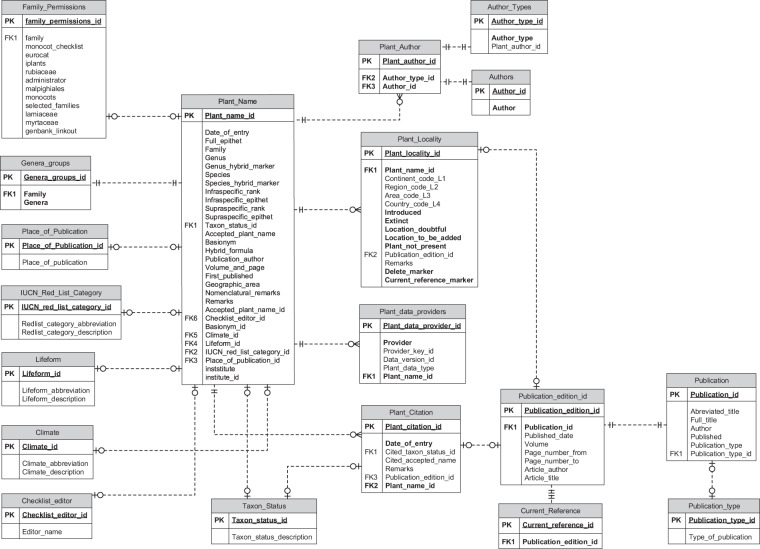
Normalised relational data model showing data structure of World Checklist of Vascular Plants.

Within the compilation system, the master version of each data record is stored in the normalised relational data model depicted in Fig. 1.

The data records are entered and maintained in this data model through a Microsoft Access front end application which links to a Sybase ASE 15.1 database via ODBC linked tables. Because ASE 15 is now unsupported, plans are being made to move the database to MariaDB or Microsoft SQL Server. Both the Sybase database and Access frontend editing tool are currently hosted internally on Kew’s servers.

The WCSP web portal used Java Servlet technology and queried the master Sybase database directly via JDBC. The new WCVP web portal is currently hosted on Google Cloud infrastructure and uses a Solr index to store and serve all the WCVP name data. The Solr index is refreshed regularly using a flat, pipe ‘|’ delimited text dump of the names data stored in Sybase and these names data are available for all users to download through the WCVP web portal at (https://wcvp.science.kew.org/).

The WCVP data download is also available Kew’s FTP server (http://sftp.kew.org/pub/data-repositories/WCVP/) where users can download the latest and previous versions of the WCVP data. So far four downloads have been made available, with the latest being from March 2021. Users can download a ‘.zip’ file which contains a text delimited file of all the names in WCVP. Once downloaded and extracted the text file is in ‘UTF-8’ format and the text delimiter is the pipe ‘|’ character (see Table 3).

A new data platform is planned to facilitate data curation and is envisaged to include an Application Programming Interface (API) to enhance third party access to and interaction with the data. In the short-term, an R interface has been developed replicating the functionality of the WCVP webpage and is currently being tested.

## Technical Validation

The processes which provide assurance of the technical rigour of the content entered and edited in the WCVP can be divided into those related to nomenclature and those concerning taxonomy.

Botanical nomenclature follows strict rules as set out by the ICN and therefore automated checks can be easily done to check compliance of WCVP content with the requirements of the ICN. We undertake checks to ensure that: (1) every name has an author; (2) every combination has the parenthetical author matching the basionym author; (3) all homotypic names refer to the same accepted or unplaced name; (4) all names of infraspecific rank have an infraspecific epithet and (5) duplicate records which came to light when IPNI LSIDs were added have been removed.

Although taxonomic decisions are subjective, they nonetheless create an underlying structure which can be checked through automated processes. We check that: (1) all names are categorized either as accepted, or as a synonym, or as an unplaced name; (2) all accepted species names belong to an accepted genus; (3) all accepted genera belong to an accepted family; (4) all synonyms refer to an accepted name; (5) all accepted names have a WGSRPD level 3 code.

## Usage Notes

While all of the information provided above is potentially useful for researchers wishing to use WCVP, in this section we focus on the questions which are most frequently asked of the compiler.

1. What are unplaced names?
  A name is unplaced if it cannot be accepted but it has not been established as a synonym. The main reasons why a name cannot be accepted are: (1) the name is not in accordance with the ICN; (2) the genus to which the name belongs is not accepted; (3) no correct name for the species exists in the genus to which the species concept belongs; (4) the name does not belong to a species concept. These latter names unrelated to species concepts are largely 19^th^ century names whose identity could not be established. The main reasons a name cannot be established as a synonym are: (1) no type exists; (2) the type has been destroyed; (3) the type is missing; (4) no scientist has studied and published on the type specimen.
2. Why is this name a synonym?
  The main reasons why a name is listed as a synonym are (1) it is published as such; (2) experts advise us that it is a synonym; (3) a determination on the type specimen indicates that it is a synonym; (4) it is an infraspecific name not in current use; (5) the name refers to a cultivar of the species.
3. Why are my newly published names missing?

Newly published names are added to WCVP from IPNI each year and go through an editing phase before being made available online, so it may take up to two years from their original publication to their inclusion in WCVP online.

## Data Availability

The software was developed and deployed using licensed third-party products (Microsoft Access and Sybase), so the code cannot be shared or be considered Open Source. Readers wishing to request access to or discuss the software currently in place, should use the contact address on the ‘Feedback’ page on the WCVP website https://wcvp.science.kew.org/feedback.

## Online-only Table

**Online-only Table 1:** Examples of peer-reviewed academic research publications using WCVP as a source of data supporting analyses in a wide diversity of disciplines.

Title^(Ref)^	Description	Discipline
Areas of plant diversity – what do we know?^34^	Review of geographical patterns of plant diversity around the world, comparison with vertebrate groups and identification of next steps for better characterising diversity patterns to achieve effective conservaion prioritization.	Biodiversity, Biogeography, Conservation
Extinction risk and threats to plants and fungi^35^	Illustrates how understanding current & projected threats to plants and fungi is necessary to manage and mitigate risks, while building awareness of gaps & bias in assessment coverage is essential to prioritize conservation efforts.	Conservation, Extinction risk, Biodiversity
EpiList 1.0: a global checklist of vascular epiphytes^36^	First global list of vascular epiphytes based on standardized definitions and taxonomy	Biodiversity, Ecology,
New Guinea has the world’s richest island flora^37^	New Guinea has the world’s richest island flora. Expert-verified checklist of the vascular plants of mainland New Guinea and surrounding islands.	Botany, Taxonomy, Ecology, Biodiversity
Historical legacies and ecological determinants of grass naturalizations worldwide^38^	Understanding geographic differences in knowledge of plant extinction, species occurrence knowledge through comparison of grasses (Poaceae) of Madagascar and the British Isles	Botany, Taxonomy, Ecology, Extinction, Biodiversity, History
Inequality in plant diversity knowledge and unrecorded plant extinctions: An example from the grasses of Madagascar^39^	Used national and subnational checklists of native and introduced grass species to determine the extent to which each region was a source or recipient of exotic grass species. Asked how species traits may distinguish those grass species that have naturalized outside their native range from those that have not, and how environmental conditions are related to the distribution of exotic grass species.	Botany, Taxonomy, Ecology, Invasive Plants, Weeds, Biodiversity, History
Vulnerability to climate change of islands worldwide and its impact on the tree of life^40^	Study aim was: (i) to map the relative vulnerability of islands to each of these threats from climate change on a worldwide scale; (ii) to estimate how island vulnerability would impact phylogenetic diversity.	Botany, Taxonomy, Ecology, Biodiversity, Conservation, Climate,
Gap analysis of Indonesian priority medicinal plant species as part of their conservation planning^41^	Identification of gaps in current in and *ex situ* conservation of Indonesian medicinal plant species. Recommendations for establishment of conservation reserves for priority plants in areas of Indonesia with greatest diversity of species.	Conservation, Medicine
Quinone diterpenes from *Salvia* species: chemistry, botany, and biological activity^42^	Analysis of diterpenoids from 130 *Salvia* species of central Asia/Mediterranean, eastern Asia, and Central & South America. Information on phytochemistry & biological activities (anti-cancer, antioxidant, anxiolytic & antidepressant, anti-obesity, antinflammatory), as well as antimicrobial and toxicological aspects of these compounds was provided.	Medicine, Taxonomy, Chemistry, Biological Activity
Global dataset shows geography and life form predict modern plant extinction and rediscovery^43^	Global analysis of modern extinction in plants. Almost 600 species have become extinct, at higher rates than background extinction; almost as many have been erroneously declared extinct, then rediscovered. Reports of extinction on islands, in the tropics and of shrubs, trees or species with narrow ranges are least likely to be refuted by rediscovery.	Extinction, Botany, Taxonomy, Ecology, Biodiversity, History
Nomenclatural changes in *Coleus* and *Plectranthus* (Lamiaceae): a tale of more than two genera^44^	Taxonomic changes following recircumsciption of a medicinally and horticulturally important group. Full synonomy & geographical details were provided. WCVP data were the basis for sampling for phylogenetic analysis and for revised compilation.	Horticulture, Medicine, Taxonomy
Conflicting phylogenetic signals in genomic data of the coffee family (Rubiaceae)^45^	Phylogenetic analyses on two plastid data sets and on a nuclear rDNA data set. Identification and interpretation of conflicting phylogenetic signals in the data.	Evolutionary history, Phylogenomics
